# Ascorbic Acid Ameriolates Liver Damage by Myeloperoxidase Oxidative Products in a Hamster Model of Amoebic Liver Abscess

**DOI:** 10.3389/fcimb.2022.855822

**Published:** 2022-03-22

**Authors:** Andrea Cruz-Baquero, Rosa Adriana Jarillo-Luna, Luz María Cárdenas-Jaramillo, Maria Elisa Drago-Serrano, José de Jesús Serrano-Luna, Judith Pacheco-Yépez

**Affiliations:** ^1^Bacteriología, Facultad de Ciencias de la Salud, Universidad Colegio Mayor de Cundinamarca, Bogotá, Colombia; ^2^Coordinación de Ciencias Morfológicas, Escuela Superior de Medicina, Instituto Politécnico Nacional, Ciudad de México (CDMX), México; ^3^Departamento de Sistemas Biológicos, Universidad Autónoma Metropolitana Unidad Xochimilco, Ciudad de México (CDMX), México; ^4^Deparmento de Biología Celular, Centro de Investigación y Estudios Avanzados del Instituto Politécnico Nacional, Ciudad de México (CDMX), México; ^5^Sección de Estudios de Posgrado e Investigación, Escuela Superior de Medicina, Instituto Politécnico Nacional, Ciudad de México (CDMX), México

**Keywords:** *Entamoeba histolytica*, amoebic liver abscess, hamster, myeloperoxidase, ascorbic acid

## Abstract

*Entamoeba histolytica* is a protozoan-pathogen-causing amoebic liver abscess (ALA). After amoeba establishment in the liver, it causes abundant infiltrate of neutrophils. Liver tissue damage by neutrophils results in part from anti-amoebic oxidative intermediates, including reactive oxygen species (ROS), reactive nitrogen species (RNS), and hypochlorous acid (HOCl), derived from the myeloperoxidase (MPO) enzyme. Ascorbic acid (ASC) is an antioxidant that acts as a scavenger for ROS and NOS-derived free radicals. No previous information regarding the effect of ASC concerning the participation of MPO in an experimental model of ALA in hamsters has been reported. Thus, the aim of the present work was to analyze the effect of ASC on acute ALA development and to measure the activity and gene expression of the MPO enzyme. Hamsters were treated with ASC (800 mg/kg) and then intrahepatically inoculated with *E. histolytica* trophozoites. Animals were sacrificed at 3, 6, and 12 h post-inoculation (p.i.), and liver samples were collected. The percentage of lesions, amoeba *in situ* count, MPO activity, and *mpo* gene expression were ascertained. Compared to ALA hamsters without ASC treatment as the control group (CT), the ALA group treated with ASC had a significant decrease in liver lesions (all p.i. hours) and viable amoeba count (12 h p.i.) and an increase in MPO activity (12 h p.i.) and *mpo* gene expression (6 h/12 h p.i.). These data suggest that ASC ameliorated liver damage caused by oxidizing products *via* modulation of *mpo* expression and activity.

## Introduction

Amoebiasis is an intestinal infection caused by the pathogen *Entamoeba histolytica (E. histolytica).* This species of amoeba can invade the intestine and then disseminate to target organs such as the liver and form amoebic liver abscesses (ALA) (Brandt and Tamayo, 1970). As documented in hamsters as an animal model of experimental ALA, during the acute phase, massive neutrophil infiltration takes place; the neutrophils surround the amoebic trophozoites to isolate them and block them from making contact with the hepatic parenchyma (Tsutsumi et al., 1984; Campos-Rodríguez et al., 2016).

The acute inflammatory response entails extensive leukocyte lysis causing, in part, liver parenchyma damage (Tsutsumi et al., 1984; Olivos-Garcia et al., 2004). Hepatic damage presumably results from the neutrophil release of ROS and RNS involved in oxidative mechanisms and hypochlorous acid (HOCl), which participates in oxidative mechanisms (Denis and Chadee, 1989; Velazquez et al., 1998; Ackers and Mirelman, 2006; Pacheco-Yépez et al., 2011; Cruz-Baquero et al., 2017). Due to their oxidative action, ROS and RNS display antimicrobial effects that also entail host cell damage by provoking peroxidation of phospholipids and proteins, allowing increased membrane permeability and, therefore, cell lysis (Kumar and Sharma, 2010).

Activated neutrophils produce cytokines that stimulate the release of ROS and increase degranulation (Shepherd and Hoidal, 1990). Neutrophils oxidative bursts yield superoxide anion $\left({\text{O}}_{2}^{-}\right)$ through nicotinamide adenine dinucleotide phosphate oxidase (NOX). ${\text{O}}_{2}^{-}$ is converted to hydrogen peroxide (H_2_O_2_) by superoxide dismutase enzyme (SOD). Ultimately, the H_2_O_2_ acts as a substrate for myeloperoxidase (MPO) to produce HClO (Hampton et al., 1998; Nathan, 2006; Tseng and Liu, 2014). MPO is a cationic enzyme located in azurophilic granules (Klebanoff, 2005). Neutrophil degranulation results in the release of MPO, which binds monocytes, stimulating the production of ROS (Shepherd and Hoidal, 1990; Kumar and Sharma, 2010). MPO oxidizes chloride ions to yield HOCl, a highly cytotoxic molecule for amoebae. Previous *in vitro* assays by our group showed that MPO binds to the *E*. *histolytica* surface and kills amoebic trophozoites, suggesting that MPO may play an important role in the innate immune response against this parasite (Pacheco-Yépez et al., 2011).

Experimental settings have focused attention on ascorbic acid (ASC) due to its antioxidant free radical scavenging activity and its presumably immunomodulatory protective role (Kaźmierczak-Barańska et al., 2020).

Previous studies indicated that ASC might alleviate or prevent infections caused by bacteria, viruses, and parasites (Hamilä and Louhiala, 2013; Hamilä and Koivula, 2013; Hamilä, 2017; Shi et al., 2021).

A protective effect of ASC against protozoan infections has been previously reported in mice infected with *Leishmania infantum* (Kato et al., 2014) or *Tripanosoma cruzi* (Castanheira et al., 2018). Probably, ascorbic acid administration will result in a change in the hepatic parenchyma damage, the number of viable amoebae, the resolution of the ALA, and the MPO enzyme alteration. The hypotheses of the present work are the antioxidant activity of ASC and the possible regulatory effect of the MPO on avoiding the ALA acute development. The data suggested a protective role of ASC against ALA by both reducing liver tissue damage and contributing to amoebic trophozoite killing.

## Materials and Methods

### Animals

This study was conducted in male golden hamsters (*Mesocricetus auratus*), with 100 g body weight. The animals were handled in accordance with Mexican federal regulations for animal experimentation and care (NOM-062-ZOO-1999, Ministry of Agriculture, México City, México). The current protocol was developed based on the ARRIVE guidelines for reporting animal research and was approved by the Ethics in Research Committee of the Escuela Superior de Medicina, Instituto Politécnico Nacional (IPN). All hamsters used in this study were handled in accordance with the guidelines of the 2000 AVMA Panel of Euthanasia.

### Amoeba Axenic Cultures

Trophozoites of *E. histolytica* strain HMI : IMSS were cultured axenically in Diamond trypticase yeast iron extract (TYI-S-33) at 37°C, as previously described (Diamond et al., 1978). The inoculum was prepared from 48 h amoebic cultures by chilling to 4°C (Contis Montes de Oca et al., 2020).

### Production of ALA and Tissue Collection

Hamsters were randomly distributed into two infected groups: an experimental group treated with ASC and a control group (CT). Animals received ASC (800 mg/kg of weight) by the orogastric route every 24 h for 3 days prior to amoeba inoculation. Hamsters were then inoculated by the intrahepatic route with 1 × 10^6^ *E*. *histolytica* trophozoites. Animals were euthanized at 3, 6, and 12 h of ALA development with sodium pentobarbital (0.5 mg/100 g) (Atitalaquia, Hgo, México). The organ was removed and weighed; then, the lesions were dissected, and their weight was quantified to obtain the percentage of damaged parenchyma. After this, fragments of lesions and healthy areas were fixed with 4% of paraformaldehyde for histological and immunochemistry analysis; additional liver samples were immediately frozen at −80°C in phosphate-buffered saline (PBS) pH 7.2 until use in MPO activity assays or in TRIzol (Ambion, Van Way, Carlsbad, CA, USA) for real-time PCR (qRT-PCR) *mpo* expression assays. All procedures were conducted according to the methods previously described in detail (Pacheco-Yépez et al., 2011; Cruz-Baquero et al., 2017; Contis Montes de Oca et al., 2020).

### Histological and Quantitative Evaluation of Viable Amoebae

Liver tissues fixed with 4% paraformaldehyde in PBS were dehydrated, embedded in paraffin, sliced into 7-μm sections, and stained with hematoxylin–eosin (Pacheco-Yépez et al., 2011). Damaged and undamaged amoebae were quantified in ALA by examining their morphological changes/alterations in the lesion areas (n = 5). Quantification was performed by using Image-Pro Plus 5.1 software (Media Cybernetics, Inc., Rockville, Maryland, USA) under a microscope (Nikon, Eclipse E-600, Tokyo, Japan) at 40× magnification (Cruz-Baquero et al., 2017).

### Immunolocalization of MPO-Positive Cells in ALA

For immunohistochemistry, liver tissue paraffin sections were mounted on glass slides. After deparaffinization and rehydration, the samples were chilled and washed with citrate buffer for 20 min and then with PBS. Endogenous peroxidase activity was blocked by incubation with 3% H_2_O_2_ in PBS for 30 min at room temperature (RT). Nonspecific reactions were blocked by treatment with 3% fetal bovine serum in PBS for 1 h at RT. The slides were incubated with D avidin solution (Vector Laboratories, CA, USA), washed with PBS, and incubated with biotin solution for 15 min. Sections were incubated overnight at 4°C with 1 μg/ml rabbit polyclonal anti-MPO (Abcam, Cambridge, UK). The samples were then incubated with a biotin-goat anti-rabbit IgG H+L DS grade secondary antibody, (Invitrogen, ZYMED, CA, USA) at 3 μg/ml for 1 h, followed by incubation with streptavidin–biotin peroxidase (Invitrogen ZYMED, CA, USA) at 2.5 μg/ml for 1 h. Diaminobenzidine (Kit DAB Pierce, IL, USA) and H_2_O_2_ as substrate were added. The sections were counterstained with hematoxylin (1:9), dehydrated, and analyzed by microscopy (NIKON Eclipse E600, Tokyo, Japan) (Cruz-Baquero et al., 2017).

### MPO Chlorination Activity

MPO activity was determined as follows: fragments of ALAs were homogenized in PBS and sonicated. Subsequently, the samples were centrifuged at 10,000×*g* at 4°C for 15 min. The supernatant was removed and then stored at −80°C until use. Myeloperoxidase chlorination assays were performed following the manufacturer’s recommendations (cat. no. 10006438, Cayman Ann Arbor, MI, USA). The results were read with a 96-well fluorescence reader (Biotek Synergy, Winooski, VT, USA). Fluorescence was analyzed with an excitation wavelength of 480−495 nm and an emission wavelength of 515−525 nm to determine relative fluorescence units (RFUs). To obtain the MPO activity, the equation previously reported was applied (Malle et al., 2007; Cruz-Baquero et al., 2017 and Podrez et al., 2000).

### MPO Quantitative Real-Time PCR

Expression of the *mpo* gene in both experimental groups was determined in total RNA from ALA. Briefly, total RNA was extracted with the TRIzol method (TRIzol, Ambion, Van Way, CA, USA). RNA quantification was carried out using a Nanodrop Lite (Thermo Scientific, MA, USA). cDNA was synthesized according to previously reported methods (Cruz-Baquero et al., 2017). Specific oligonucleotide primers were generated with online assay design software (probeFinder: http://www.universalprobelybrary.com). Expression of the *mpo* gene in hamsters (Gene iD: 101830171) was determined by utilizing a prediction of the *mpo* gene sequences found in the NCBI database. The sequence of the hamster *gapdh*, used as an internal control (Gene ID: 106022412), was taken from the same database. Specific oligonucleotide primers were generated using the aforementioned method, and the primer sequences employed for hamsters were *mpo*F 5′ GCTGTGCACT GAACACACCT3′ and *mpo*R 5′ TTTAGGAAGCTACGGGATGG′. The reaction mixture was used as previously described (Cruz-Baquero et al., 2017). qRT-PCR was carried out in a LightCycler 1 Nano SW 1.1 Instrument (Roche Diagnostics, IN, USA), and data were analyzed with LightCycler software. mRNA levels were calculated by the comparative parameter quantification cycle method with the reference gene *gapdh* (Cruz-Baquero et al., 2017).

### Statistical Analysis

All experiments were performed at least three times independently. The results were expressed as means ± SD. The comparison between two groups was analyzed using Student’s unpaired two-tailed *t-test*. If a significant (*p*<0.05) main effect or association was identified, the respective group means were compared using the Bonferroni test. All analyses were performed with the GraphPad Prism software version 6 (Systat Software Inc., San Jose, CA, USA).

## Results

### Effect of Ascorbic Acid Treatment on ALA Histopathology

**Figure 1:**
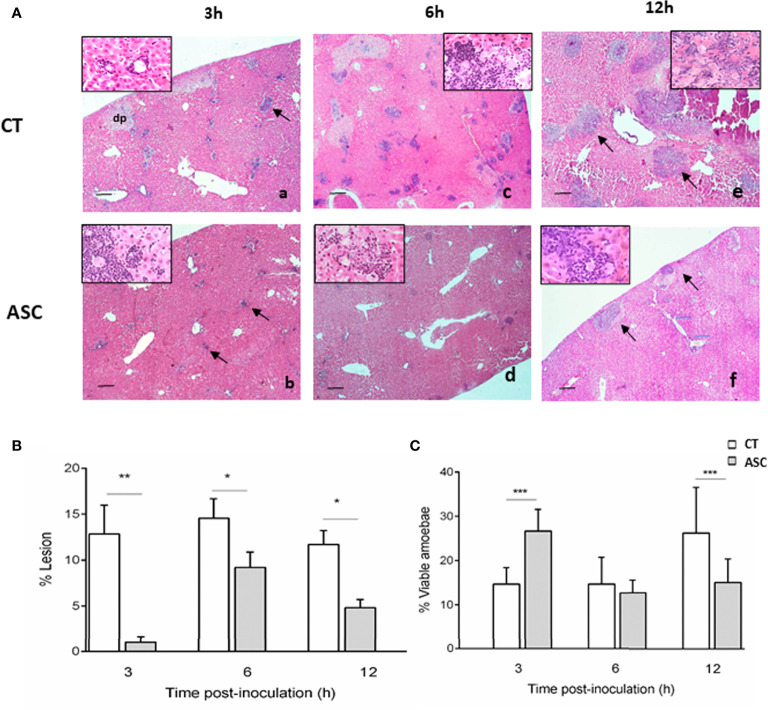
**(A)** Histological analysis of hamsters inoculated with amoebae and treated (Ab, Ad, Af) or not with ASC (Aa, Ac, Ae). (Aa) ALA inflammatory infiltrate (arrow) and area of damaged parenchyma and the presence of damaged amoebae (inserted box). (Ab) Animals treated with ASC showed inflammatory foci (arrows) with the presence of amoebae (inserted box). (Ac) ALA 6 h, presence of trophozoites (inserted box). (Ad) Animals treated with ASC showed inflammatory foci and amoebae (inserted box). (Ae) ALA 12 h large necrotic area with the presence of amoebae (inserted box) and inflammatory cells (arrows). (Af) Animals treated with ASC at 12 h showed less damage to the parenchyma and inflammatory infiltration (arrows) and damaged amoebae (inserted box). Bar = 200 μm. The liver was removed and weighed to determine the percentage (%) of liver damage in ALA using the following formula: % ALA = (abscess weight/total liver weight) × 100. The percentage of ALA was significantly diminished at 3, 6, and 12 h p.i. in hamsters treated with ASC compared with CT hamsters **(B)**. The percentage of viable amoebae was higher in the ASC group at 3 h, similar in the two groups at 6 h and decreased in the ASC group at 12 h **(C)**. ***p < 0.001, **p < 0.01, *p < 0.05.

Hematoxylin–eosin staining of ALA at 3, 6, and 12 h p.i. in hamsters treated or not treated with ASC showed inflammatory infiltrates constituted by polymorphonuclear leukocytes, mainly neutrophils (**Figure 1A**). At 3 h p.i., the animals treated with ASC presented smaller parenchymal lesions (**Figure 1Ab**), and the percentage of viable amoebae was higher in animals treated with ASC compared with CT group (**Figure 1Ab**, inserted box vs. 1Aa inserted box). At 6 h p.i., the area of damaged parenchyma was increased; however, while it was smaller in the animals that received ASC (**Figure 1Ad**), the size of their inflammatory infiltrate was similar between the groups (**Figures 1Ac, Ad**). The number of viable amoebae was similar between the animals that received ASC and CT (**Figures 1Ac, Ad**, inserted box). At 12 h p.i., the area of damaged liver parenchyma continued to be greater in CT animals (**Figure 1Ae**) than in the ASC group (**Figure 1Af**); however, the percentage of viable amoebae was lower in the latter group (**Figure 1Af** vs. **1Ae**, inserted box).

### Outcome of Ascorbic Acid Treatment in ALA Lesions and Viable Amoebae

The percentage (%) of ALA at 3, 6, and 12 h was determined. ASC induced a significant decrease in the percentage of ALA lesions at 3, 6, and 12 h p.i. compared to the CT group (**Figure 1B**). Compared to the CT group, the percentage of lesion was significantly lower in the ASC group at 3 h (p<0.01), 6 h, and 12 h (p<0.05) (**Figure 1B**). Additionally, in the ASC group, the percentage of viable amoebae in ALA was increased at 3 h p.i. (p<0.001) similar to that at 6 p.i. and decreased significantly at 12 h p.i. with regard to the CT group (p<0.001) (**Figure 1C**).

### Outcome of Ascorbic Acid Treatment on MPO-Positive Cells in ALA Lesions

**Figure 2:**
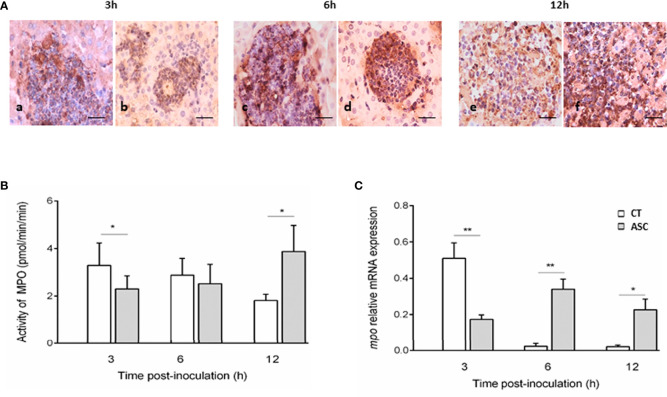
Immunohistochemistry of MPO in inflammatory infiltrates **(A)**. The inflammatory cells present in the lesions of the CT (Aa, Ac, Ae) and ASC (Ab, Ad, Af) animals showed a positive reaction to MPO at the three times studied. Bar = 40 μm. At 3 h, MPO activity was lower in the ASC group than that in the CT group; at 6 h, the activity was similar in both groups, and at 12 h, MPO activity was greater in the ASC group than that in the CT group **(B)**. **(C)** *mpo* expression was significantly increased in hamsters treated with ASC compared with the CT group at 6 and 12 h or decreased at 3 h p.i. **p < 0.01, *p < 0.05.

We evaluated the *in situ* presence of MPO enzyme in ALA lesions from hamsters inoculated with amoebae and treated or not with ASC. In the CT and ASC groups at all times studied (3, 6, and 12 h), the inflammatory infiltrates showed cells with a positive staining for MPO (**Figures 2Aa–Af**).

### Ascorbic Acid Increased the MPO Enzyme Activity in ALA

MPO activity was determined in hamsters inoculated with *Entamoeba* trophozoites and treated or not with ASC. At 3 h of ALA evolution, MPO activity was lower in the ASC group than in the CT group (p<0.05). At 6 h p.i., no differences in MPO activity between the CT and ASC groups were observed. At 12 h p.i., the MPO activity increased significantly in ASC animals compared with the CT group (p<0.05) (**Figure 2B**).

### Expression of the *mpo* Gene Increased in ALA of Hamsters Inoculated With Ascorbic Acid

The gene *mpo* gene expression level was significantly increased at 6 (p<0.01) and 12 h p.i. (p<0.05) in the ASC-treated group relative to the CT group. At 3 h of ALA development, *mpo* gene expression was lower in the ASC group than in the CT group (p<0.01) (**Figure 2C**).

## Discussion

Previous studies have indicated that ASC may alleviate or prevent infections caused by bacteria, viruses, and parasites (Hamilä and Louhiala, 2013; Hamilä and Koivula, 2013; Hamilä, 2017; Shi et al., 2021). ASC is considered the most important water-soluble antioxidant, and it acts under conditions where oxidative stress is elevated. ASC can easily scavenge peroxyl radicals, superoxide anions, singlet oxygen, and hypochlorite (Sies and Stahl, 1995).

Our hypotheses entailed that ASC administration would result in (i) the hepatic parenchyma damage modification, (ii) the number of viable amoebae, (iii) the ALA resolution, and (iv) the MPO enzyme alteration. In this study, for the first time, it is reported that ASC induces histopathological changes in ALA evolution, as evidenced by the reduction in both hepatic parenchyma damage and the number of viable amoebae. As we hypothesized, the percentage of lesion of hamsters treated with ASC was smaller than CT hamsters; the viable amoebae percentage in ALA hamsters was lower (at 12 h p.i.) with ASC treatment. As we expected, the changes in the percentages of viable trophozoites are related to the kinetics of the expression and activity of MPO observed in the hamsters treated with ASC.

A pioneering work by Tsutsumi et al. (1984) demonstrated that the extent of necrosis in the acute stage of ALA hepatic parenchyma is attributable to massive lysis of leukocytes, prominently neutrophils, and the release of oxidative products generated by MPO in these leukocytes. Here, the histological analysis demonstrated that animals inoculated with *E*. *histolytica* and treated with ASC showed damaged trophozoites, as evidenced by lytic spaces, compared with the CT group; this finding showed that ASC changed the overall outcome of the early stages of ALA. In our study, the ASC diminished the ALA percentage, implying that ASC might have an effect on the resolution of ALA in the acute phase. No previous work on ALA and ASC has been reported; however, in other intracellular parasitosis, the effect of ASC has been evaluated. Shi et al. (2021) demonstrated that ASC inhibited *Plasmodium berghei* and *Plasmodium falciparum* growth and that ASC absorption causes the accumulation of ROS in red-blood-infected cells, inducing the apoptosis of these parasites. In addition, Puente et al. (2018) reported that ASC has a lethal pro-oxidant effect on the *T. cruzi*, and *in vivo*, the parasites number in murine Chagas’s disease model was lower than that in the CT group.

We also observed a significant reduction in the percentage of viable amoebae in animals inoculated with *E*. *histolytica* and treated with ASC. The antioxidant effect of ASC may explain the reason why the number of viable amoebae present in inflammatory infiltrates was higher in the first hours after ALA establishment, since it has been reported that *E. histolytica* is susceptible to oxidizing agents (Ghadirian et al., 1986). Thus, by inactivating the effect of HOCl, the damage to amoeba during the early phase decreased. In addition to its direct antioxidant effect, in various inflammatory processes where the participation of MPO is important, the administration of ASC alone, or in combination with other antioxidants, reduces the activity of the MPO (Zhang et al., 2007; Guerra et al., 2012; Yan et al., 2015).

Although MPO-positive cells were found in the ALA hamster *in situ* in both groups treated or not with ASC, the reduction in the size of the lesions in ASC-treated hamsters may be attributed to the antioxidant effect of ASC in reducing the oxidative damage to the hepatic parenchyma. The hepatoprotective effect of ASC has been previously reported in BALB/c mice infected with *Leishmania infantum* (Kato et al., 2014). Moreover, ASC is considered a physiological substrate for myeloperoxidase (MPO), and its effect on myeloperoxidase-dependent processes is widely attributed to its scavenger or quencher actions on HClO (Parker et al., 2011).

We also evaluated the MPO activity in ASC-treated or untreated hamsters inoculated with amoeba; our results showed that the MPO activity was low at 3 h of ALA development, a time when there were more viable trophozoites. Additionally, a significant increase in MPO activity at 12 h of ALA development in animals treated with ASC correlated directly with the decrease in the number of viable amoebae and with lesions of lesser extension. These data showed that MPO activity increased in parallel with amoebae elimination in ASC-treated animals. Moreover, a report by Marquez and Dunford (1990) demonstrated that in the presence of ASC, compound III is rapidly converted back to its native state, making the MPO enzyme available for HOCl production, the main bactericidal product of the MPO enzyme. MPO activity is not restricted to phagosomes; this enzyme can be released into the extracellular space during the inflammatory process by neutrophils as a result of incomplete phagocytosis, cell necrosis, or the formation of NETs. In our study, the release of MPO correlates with massive lysis of inflammatory cells, probably allowing HOCl-mediated damage to amoebae. A previous work of our group demonstrated that *in vitro*, MPO significantly reduced the viable amoebae (Pacheco-Yépez et al., 2011).

Furthermore, the expression of MPO increased at 6 and 12 h of ALA evolution, and this increase in *mpo* expression favored the activity of the enzyme and the resolution of ALA in the hamster model. Our results suggest that ASC has a modulating effect on the activity of MPO and that, together with its antioxidant effect, it has a protective effect against ALA in hamsters.

However, additional studies with longer follow-up are necessary to evaluate the beneficial effect of ASC and explore other effects of ASC, such as its stimulating effect on the activity of antioxidant enzymes reported in other conditions of inflammation, and to elucidate other mechanisms of action.

## Data Availability Statement

The original contributions presented in the study are included in the article/supplementary material, further inquiries can be directed to the corresponding author.

## Ethics Statement

The animal study was reviewed and approved by Comité de Investigación de Cuidado y Uso de Animales de Laboratorio (ESM-CICUAL-04/2006-08-2011), Escuela Superior de Medicina, Instituto Politécnico Nacional.

## Author Contributions

ACB: performed experiments, data analysis, figure preparation and participated in writing and review of manuscript. RAJL: performed experiments, sampling, histopathology count, statistical analysis of histopathology data, and figure preparation. LMCJ: liver sampling processing for histopathology observation, myeloperoxidase labeling, figure preparation, and data analysis. MEDS: data analysis, figure editing, critical manuscript reading, manuscript formatting, and editing. JJSL: drafting preparation and critical reading. JPY: original conception, experimental design, manuscript drafting, reference curation, searching, retrieving, reading, sorting, and selection. All authors contributed to the article and approved the submitted version.

## Funding

This study was supported by Consejo Nacional de Ciencia y Técnología (Grant/Award Number: 181566) and Secretaria de Investigación y Posgrado, Instituto Politécnico Nacional (Grant/Award Number: 20190288).

## Conflict of Interest

The authors declare that the research was conducted in the absence of any commercial or financial relationships that could be construed as a potential conflict of interest.

## Publisher’s Note

All claims expressed in this article are solely those of the authors and do not necessarily represent those of their affiliated organizations, or those of the publisher, the editors and the reviewers. Any product that may be evaluated in this article, or claim that may be made by its manufacturer, is not guaranteed or endorsed by the publisher.
